# Correction: Schupp et al. Deconstructing Intratumoral Heterogeneity through Multiomic and Multiscale Analysis of Serial Sections. *Cancers* 2024, *16*, 2429

**DOI:** 10.3390/cancers18172765

**Published:** 2026-08-26

**Authors:** Patrick G. Schupp, Samuel J. Shelton, Daniel J. Brody, Rebecca Eliscu, Brett E. Johnson, Tali Mazor, Kevin W. Kelley, Matthew B. Potts, Michael W. McDermott, Eric J. Huang, Daniel A. Lim, Russell O. Pieper, Mitchel S. Berger, Joseph F. Costello, Joanna J. Phillips, Michael C. Oldham

**Affiliations:** 1 Department of Neurological Surgery, University of California, San Francisco, San Francisco, CA 94143, USA; pschupp@sonic.net (P.G.S.); sam_shelton@hotmail.com (S.J.S.); daniel.brody@ucsf.edu (D.J.B.); reliscu@berkeley.edu (R.E.); johnbret@ohsu.edu (B.E.J.); tmazor@ds.dfci.harvard.edu (T.M.); kwkelley@stanford.edu (K.W.K.); matthew.potts@nm.org (M.B.P.); mwmcd@baptisthealth.net (M.W.M.); daniel.lim@ucsf.edu (D.A.L.); russ.pieper@ucsf.edu (R.O.P.); mitchel.berger@ucsf.edu (M.S.B.); joseph.costello@ucsf.edu (J.F.C.); joanna.phillips@ucsf.edu (J.J.P.); 2 Biomedical Sciences Graduate Program, University of California, San Francisco, San Francisco, CA 94143, USA; 3 Medical Scientist Training Program, University of California, San Francisco, San Francisco, CA 94143, USA; 4 Neuroscience Graduate Program, University of California, San Francisco, San Francisco, CA 94143, USA; 5 Department of Pathology, University of California, San Francisco, San Francisco, CA 94143, USA; eric.huang2@ucsf.edu

**Error in Figure**

In the original publication [1], there was a mistake in the published Figure 7.

In Figure 7, the **Accuracy** numbers reported in the table above the top left corner of the heat map are incorrect. They should read: 67%, 67%, and 65%.

The corrected Figure 7 appears below.

**Text Correction**

In the original publication, there was a mistake in two texts of Sections 2 and 3.

Original text 1 in Section 2.5:

“Overall, none of the algorithms for inferring malignancy from CNVs achieved accuracy > 61% (Figure 7).”

Original text 2 in Section 3: 

“In our study, we found that popular algorithms for identifying cancer cells from inferred CNVs from gene expression data [25,49,50] were only ~60% accurate.”

A correction has been made to Section 2.5 Case 2: Analysis of Gene Expression and Section 3 Discussion:

Corrected text 1 in Section 2.5:

“Overall, none of the algorithms for inferring malignancy from CNVs achieved accuracy > 67% (Figure 7).”

Corrected text 2 in Section 3:

“In our study, we found that popular algorithms for identifying cancer cells from inferred CNVs from gene expression data [25,49,50] were only ~66% accurate.”

The authors state that the scientific conclusions are unaffected. This correction was approved by the Academic Editor. The original publication has also been updated.

## Figures and Tables

**Figure 7:**
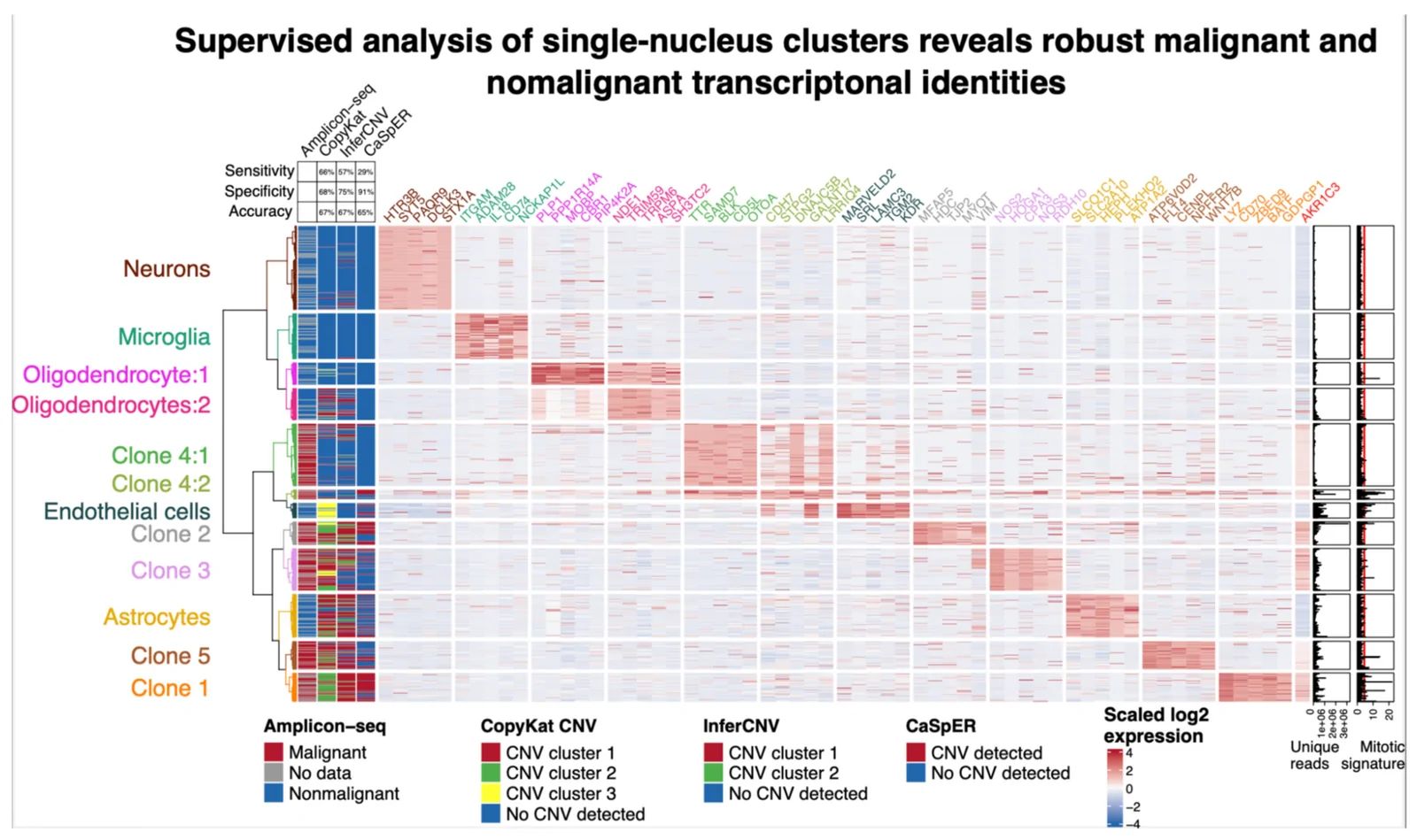
Genotyping nuclei profiled by snRNA-seq reveals the limitations of single-cell CNV-calling algorithms. Heatmap of scaled log_2_ expression vectors for the five most upregulated genes in each snRNA-seq cluster vs. all other clusters (one-sided Wilcoxon rank-sum test). Far left: malignancy vector determined by snAmp-seq of cDNA spanning mutations in the truncal clone. Left: malignancy vectors inferred from CNV analysis of snRNA-seq data using the CopyKat [49], InferCNV [25], or CaSpER [50] algorithms (blue = nonmalignant; all other colors = malignant). Right: bar plots depict the total number of unique reads (UMIs) for each nucleus and the average number of UMIs for genes comprising the Gene Ontology category ‘mitotic chromosome condensation’ (GO: 0030261). Red vertical line: max expression of mitotic genes in neurons, which presumably represents background noise.
