# Supplementary material for: Deconstructing Intratumoral Heterogeneity through Multiomic and Multiscale Analysis of Serial Sections
Source: Cancers (Basel). 2024 Jul 1;16(13):2429. doi: 10.3390/cancers16132429 (PMC11240479; doi:10.3390/cancers16132429)
Supplement: Supplementary file 1 [file cancers-16-02429-s001.zip › cancers-3051819-supplementary.pdf]

cancers

MDPI

Supplementary Figure and Table Legends

Table S1: Section usage and quality control (case 1)
Table S2: Somatic mutations identified by exome sequencing (case 1)
Table S3: Primers for amp-seq (case 1)
Table S4: Amp-seq VAFs for all tumor sections (case 1)
Table S5: CNV calls for all tumor sections (case 1)
Table S6: Cellular and clonal abundance (case 1)
Table S7: Module eigengenes (case 1)
Table S8: k_{ME} values (case 1)
Table S9: Description of gene sets used for enrichment analysis
Table S10: Gene list for group-lasso model (case 1)
Table S11: Enrichments for group-lasso model (case 1)
Table S12: Section usage and quality control (normal human brain samples)
Table S13: Module eigengenes differential coexpression
Table S14: k_{ME} differential coexpression
Table S15: Section usage and quality control (case 2)
Table S16: Somatic mutations identified by exome sequencing (case 2)
Table S17: Primers for amp-seq (case 2)
Table S18: Amp-seq VAFs for all tumor sections (case 2)
Table S19: CNV calls for all tumor sections (case 2)
Table S20: Cellular and clonal abundance (case 2)
Table S21: Single-nucleus amplicon sequencing by Mission Bio
Table S22: Module eigengenes (case 2)
Table S23: k_{ME} values (case 2)
Table S24: Gene list for lasso model (case 2)
Table S25: Enrichments for lasso model (case 2)
Table S26: Primers for snRNA-seq and snAmp-seq experiments
Table S27: snAmp-seq of snRNA-seq nuclei
Table S28: Wilcoxon rank-sum test cluster and clone associations
Table S29: Positively differentially expressed genes for snRNA-seq
Table S30: snRNA-seq cluster enrichment analysis
Table S31: Intercase correlations

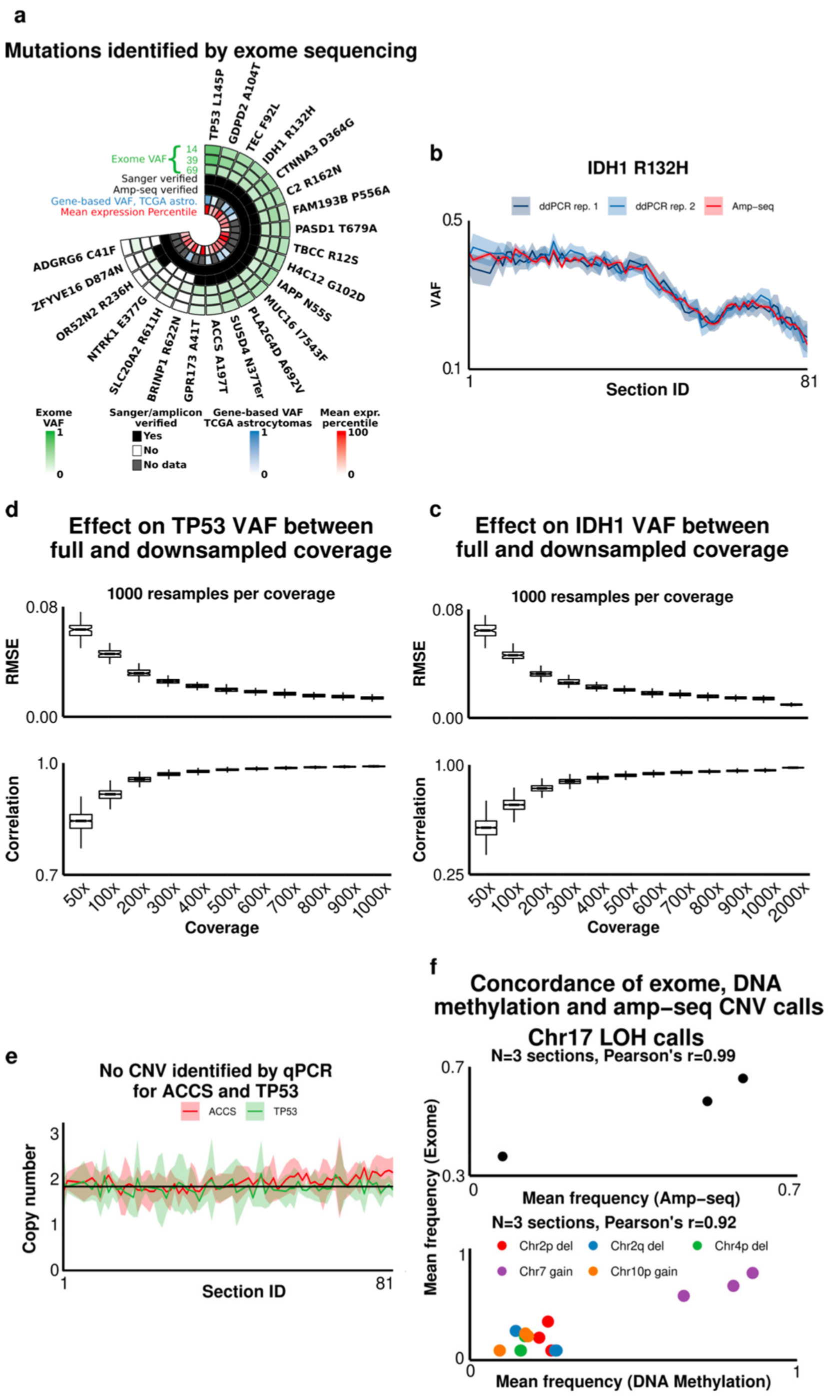

Figure S1. | Mutation validation (case 1). a) Nonsynonymous mutations were identified by exome sequencing of tumor sections 14, 39, 69, and the patient's blood. Green track: variant allele frequencies (VAF) for each mutation in each section. Black tracks: mutation validation by Sanger sequencing and amp-seq, which is more sensitive. Blue track: gene mutation frequencies in TCGA astrocytomas (n = 286). Red track: mean expression percentiles for each gene over all tumor sections. b) Amp-seq and droplet-digital PCR (ddPCR) yielded consistent estimates of IDH1 R132H variant frequencies (n = 69 tumor sections; rep. 1 and rep. 2 denote technical replicates using the same input DNA). Shaded areas represent two standard errors. c-d) Downsampling of amp-seq reads for IDH1 R132H (c) and TP53 L145P (d) was performed in each tumor section to achieve desired coverage levels (x-axis). For each downsampling (n = 1000), the root mean square-error (RMSE; top) and Pearson's correlation (bottom) was calculated with respect to the true VAF (calculated using all reads) over all sections (n = 69). e) Relative copy number was determined by SYBR Green qPCR for *TP53* and *ACCS* loci using genomic DNA from 69 tumor sections and blood. The mean of triplicate measurements, normalized to RNaseP (*RPPH1*) copy number, is shown. Shaded areas represent two standard errors.

f) Top: Concordant estimates of chr17p loss-of-heterozygosity (LOH) in the same tumor sections (n = 3) were obtained from exome data by analyzing changes in B-allele frequencies and from amp-seq data by analyzing TP53 L145P VAF, which is equivalent to chr17p LOH frequency since both events are truncal. Bottom: Concordant estimates of CNV frequencies in the same tumor sections (n = 3) were obtained using FACETS [38] and ChAMPS [39] to analyze exome and DNA methylation data, respectively.

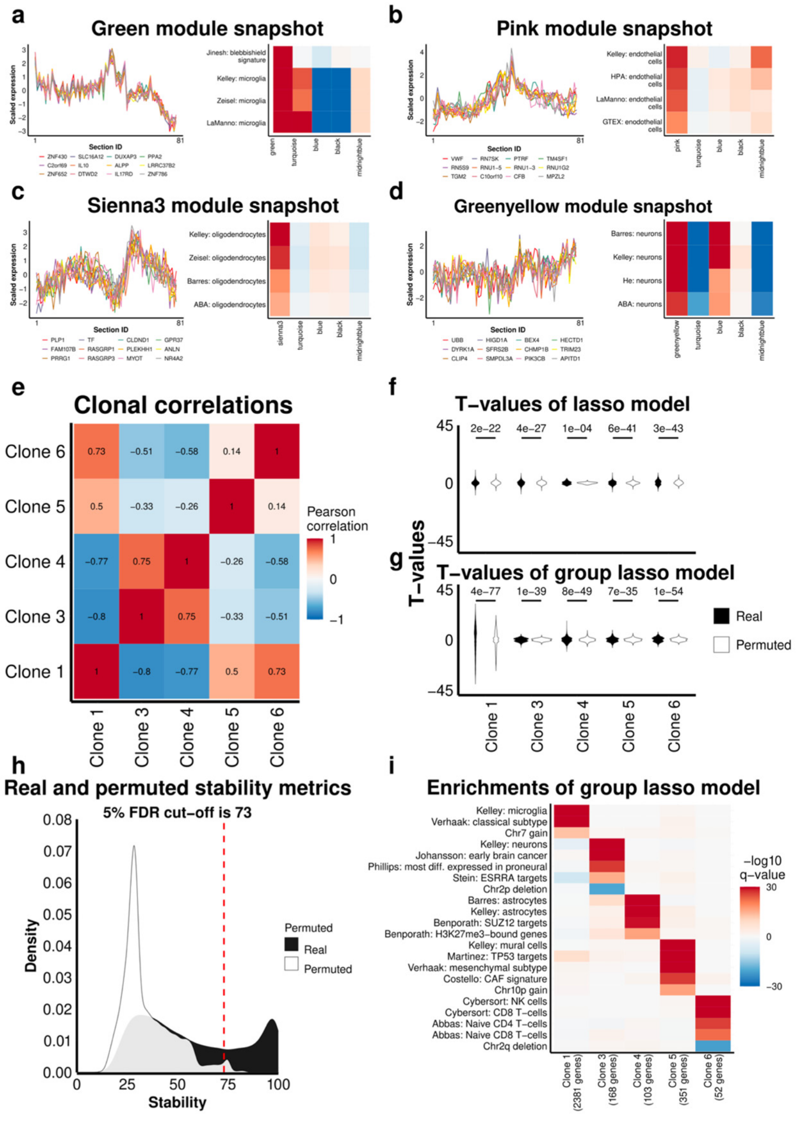

Figure S2. | Transcriptional signatures of nonmalignant cell types and malignant clones (case 1). a-d) Left: snapshots of additional gene coexpression modules enriched for markers of nonmalignant cell types (expression patterns for the top 12 genes ranked by k_{ME} are shown). Right: heatmaps of gene set enrichment results for each module. Modules included genes that were most specifically and significantly correlated (after FDR correction) to the module eigengene (ME), and enrichment was assessed with a one-sided Fisher's exact test (followed by FDR correction; see panel i for legend). e) Correlation heatmap for the cumulative frequency vectors of identified clones. f-g) Lasso regression [109] was used to model the expression of all genes (n = 20,018) as a function of clonal frequencies over all tumor sections (n = 69). Violin plots illustrate the distributions of t-values for all models where the indicated clone was the only explanatory variable that survived lasso selection. Permutations were performed by randomly scrambling clonal frequencies (n = 100) prior to lasso regression. Real and permuted clonal frequency vectors were bootstrapped (n = 100) to address collinearity. P-values denote the significance of the Anderson-Darling test, which evaluates whether two distributions are likely to be derived from the same distribution. f) Results of a standard lasso model. g) Results of a group lasso model where the truncal clone (equivalent to tumor purity) was placed in

a separate group due to its strong effect on gene expression (Figure 3c); note the general improvement in Anderson-Darling test P-values. h) Density plot showing the number of times (out of 100 bootstraps) that the same explanatory (clonal frequency vector) variable was retained by the group lasso regression model, or 'stability'. Only group lasso models where retained explanatory variables included the truncal clone and up to one other clone were considered. The vertical line demarcates the point to the right of which only 5% of values belong to the permuted distribution, i.e., a 5% FDR rate. i) Enrichment analysis (one-sided Fisher's exact test) of genes that were significantly (FDR < .05) and stably (FDR < .05) associated with each clone. Gene sets are described in Table S9. Heatmap depicts −log10 FDR-corrected p-values (q-values; shared legend for a-d) after comparing each gene set to all genes with stability > 73 for a given clone (one-sided Fisher's exact test). Positive values represent enrichments for genes with significant positive correlations to the ME (a-d) or significant positive modeling coefficients (i), while negative values represent enrichments for genes with significant negative correlations to the ME (a-d) or significant negative modeling coefficients (i).

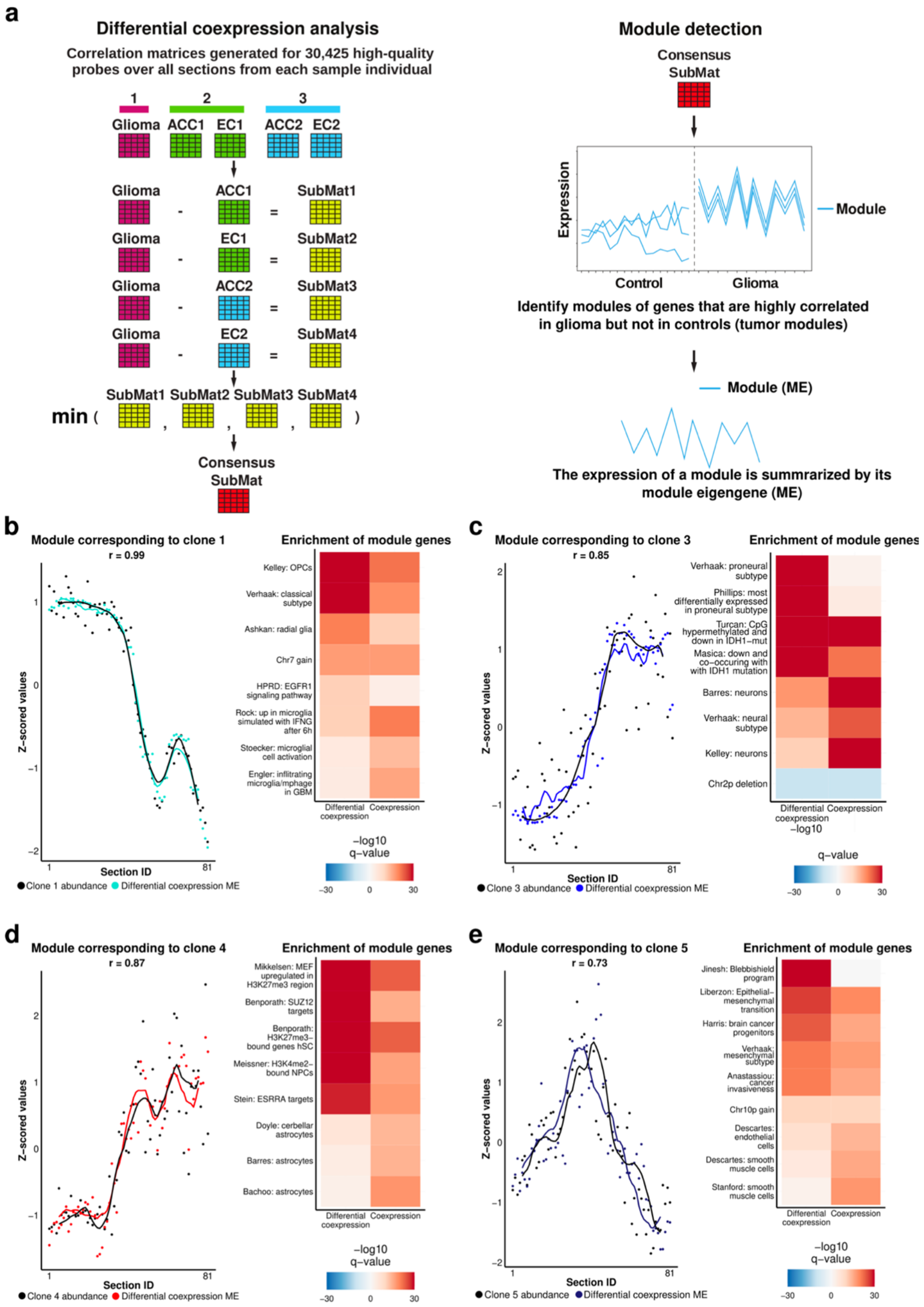

Figure S3. | Differential coexpression analysis of glioma and normal human brain preserves gene coexpression modules associated with malignant clones (case 1). a) Genome-wide gene coexpression relationships were calculated for each of the five tissue specimens (one astrocytoma and four

normal brain controls) over all tissue sections, resulting in five correlation matrices with the same dimensions. Unbiased differential coexpression analysis was performed as illustrated. ACC = anterior cingulate cortex; EC = entorhinal cortex. b-e) Left: differentially coexpressed module eigengenes (ME) with the strongest correlations to clonal abundance (defined cumulatively). Locally weighted smoothing (LOESS) lines are shown; correlation is based on data points. Right: enrichment analysis of differentially coexpressed module genes using published gene sets. FDR-corrected p-values (q-values) from one-sided Fisher's exact tests are shown. Positive values represent enrichments of genes that were significantly positively correlated to the ME, while negative values represent enrichments of genes that were significantly negatively correlated to the ME. Gene sets representing chromosomal gains or losses include all genes within affected regions (as described in Figure 2l and Table S5). See Table S9 for descriptions and sources of featured gene sets.

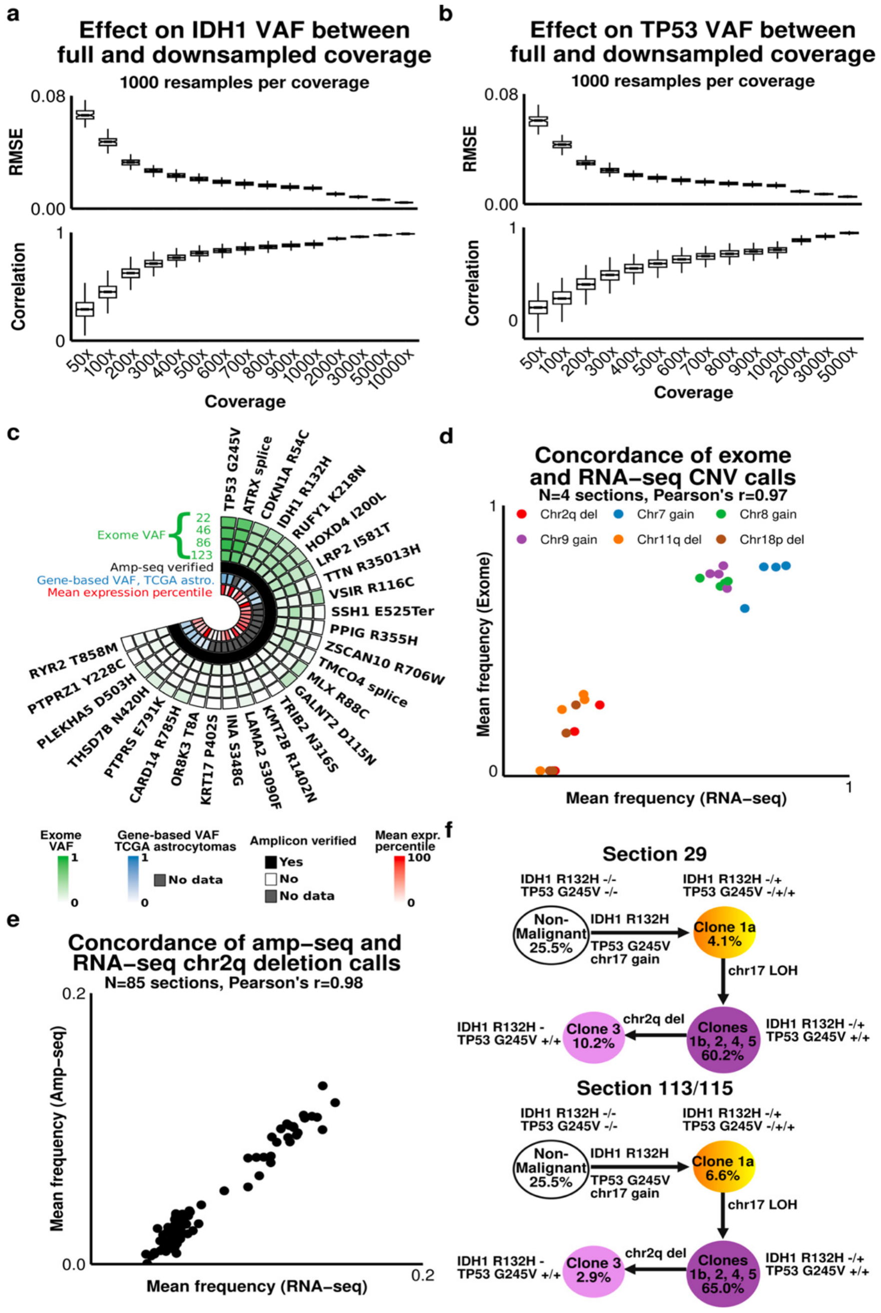

Figure S4. | Mutation validation (case 2). a-b) Downsampling of amp-seq reads for IDH1 R132H (a) and TP53 G245V (b) was performed in each tumor section to achieve desired coverage levels (x-axis). For each downsampling (n = 1000), the root mean square-error (RMSE; top) and Pearson's correlation (bottom) was calculated with respect to the true VAF (calculated using all reads) over all sections

(n = 85). c) Nonsynonymous mutations were identified by exome sequencing of tumor sections 22, 46, 85, 123, and the patient's blood. Green track: variant allele frequencies (VAF) for each mutation in each section. Black track: mutations validation by amp-seq. Blue track: gene mutation frequencies in TCGA astrocytomas (n = 286). Red track: genome-wide mean expression percentiles over all sections (n = 90). d) Concordant estimates of CNV frequencies in the same tumor sections (n = 4) were obtained using FACETS [38] and CNVkit [45] to analyze exome and RNA-seq data, respectively. e) Concordant estimates of chromosome 2q deletion frequencies in the same tumor sections (n = 85) were obtained using amp-seq (Figure 4m) and RNA-seq, which was analyzed by CNVkit. f) Clone phylogeny (with arbitrary branch lengths) derived from single-nucleus amp-seq (snAmp-seq) of mutations affecting the *IDH1* and *TP53* loci for section 29 (n = 4433 nuclei) and sections 113/115 (n = 3736 nuclei). Clone names are derived from Figure 4o, and the percentages of nuclei assigned to each clone are shown.

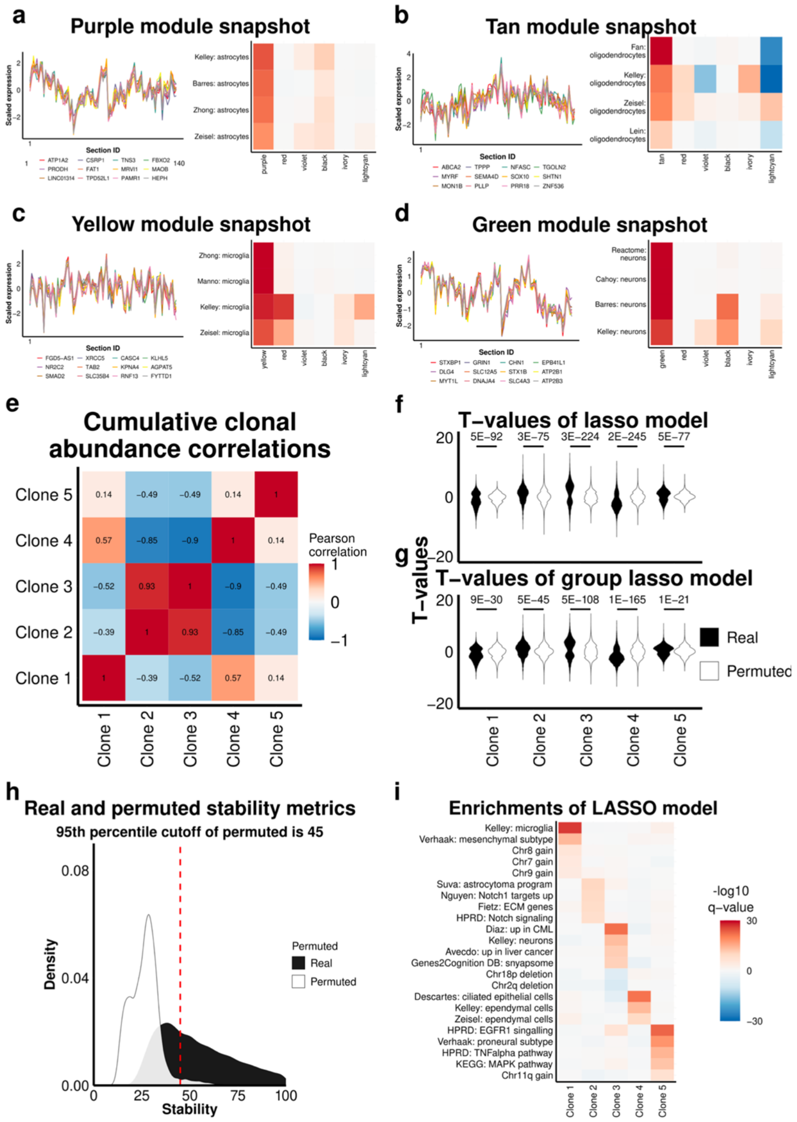

Figure S5. | Transcriptional signatures of nonmalignant cell types and malignant clones (case 2). a-d) Left: snapshots of additional gene coexpression modules enriched for markers of nonmalignant cell types (expression patterns for the top 12 genes ranked by k_{ME} are shown). Right: heatmaps of gene set enrichment results for each module. Modules included genes that were most specifically and significantly correlated (after FDR correction) to the module eigengene (ME), and enrichment was assessed with a one-sided Fisher's exact test (followed by FDR correction; see panel i for legend). e) Correlation heatmap for the cumulative frequency vectors of identified clones. f-g) Lasso regression [109] was used to model the expression of all genes (n = 20,246) as a function of clonal frequencies over all tumor sections (n = 85). Violin plots illustrate the distributions of t-values for all models where the indicated clone was the only explanatory variable that survived lasso selection.

Permutations were performed by randomly scrambling clonal frequencies (n = 100) prior to lasso regression. Real and permuted clonal frequency vectors were bootstrapped (n = 100) to address collinearity. P-values denote the significance of the Anderson-Darling test, which evaluates whether two distributions are likely to be derived from the same distribution. f) Results of a standard lasso model. g) Results of a group lasso model where the truncal clone (equivalent to tumor purity) was placed in a separate group. Unlike case 1, the group lasso model did not outperform the standard lasso model. h) Density plot showing the number of times (out of 100 bootstraps) that the same explanatory (clonal frequency vector) was retained by the standard lasso regression model, or 'stability'. The vertical line demarcates the point to the right of which only 5% of values belong to the permuted distribution, i.e., a 5% FDR rate. i) Heatmap of FDR-corrected p-values (q-values; shared legend for panels a-d) after comparing each gene set to all genes with stability > 45 for a given clone (one-sided Fisher's exact test). Positive values represent enrichments of genes with significant positive correlations to the ME (a-d) or significant positive modeling coefficients (i), while negative values represent enrichments of genes with significant negative correlations to the ME (a-d) or significant negative modeling coefficients (i).

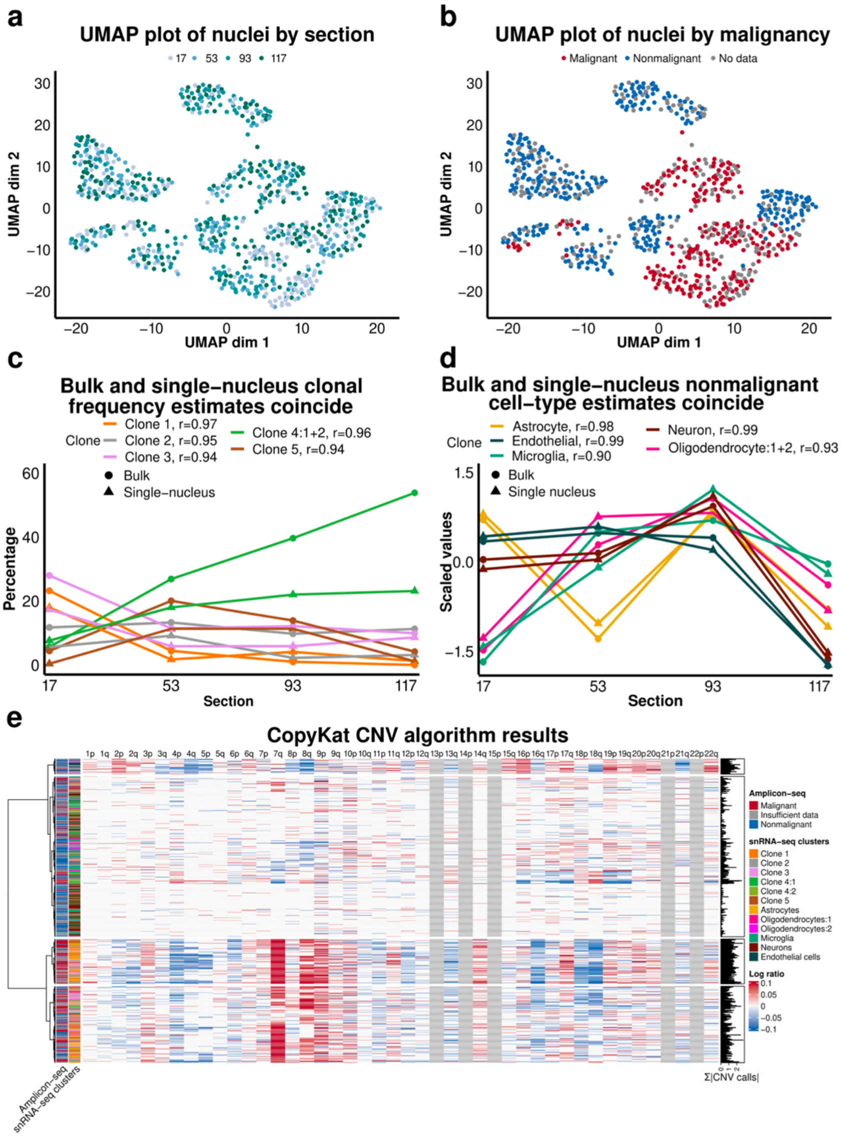

Figure S6. | Single-nucleus RNA-seq analysis validates inferences from bulk data. a) UMAP plot of snRNA-seq data (n = 809 nuclei) with the tumor section IDs that served as the source for each nucleus superimposed. b) UMAP plot of snRNA-seq data with malignancy superimposed. Malignancy was determined by genotyping all nuclei via single-nucleus amplicon sequencing (snAmp-seq) of cDNA spanning mutations in the truncal clone. c) Frequencies of malignant clones in snRNA-seq data (n = 360 nuclei from four tumor sections) and bulk data (n = 16 tumor sections), with

correlations in legend. d) Relative abundance of nonmalignant cell types in snRNA-seq data (n = 449 nuclei from four tumor sections) and bulk data (n = 16 tumor sections), with correlations in legend. Estimates were scaled and centered for comparability. Bulk estimates for (c-d) are derived from clonal abundance and module eigengene values featured in Figure 4p and Figure S5a-d, respectively, averaged across the four sections flanking each section analyzed by snRNA-seq (snRNA-seq section 17: bulk sections 14, 16, 18, 19; snRNA-seq section 53: bulk sections 50, 51, 54, 55; snRNA-seq section 93: bulk sections 91, 92, 94, 95; snRNA-seq section 117: bulk sections 114, 116, 118, 119). e) Log-ratio output of the CopyKat CNV algorithm [49]. Left: snAmp-seq malignancy assignments and snRNA-seq cluster assignments. Right: sum of the absolute value of CopyKat CNV calls (chromosomal arms in gray could not be called due to inadequate gene coverage).

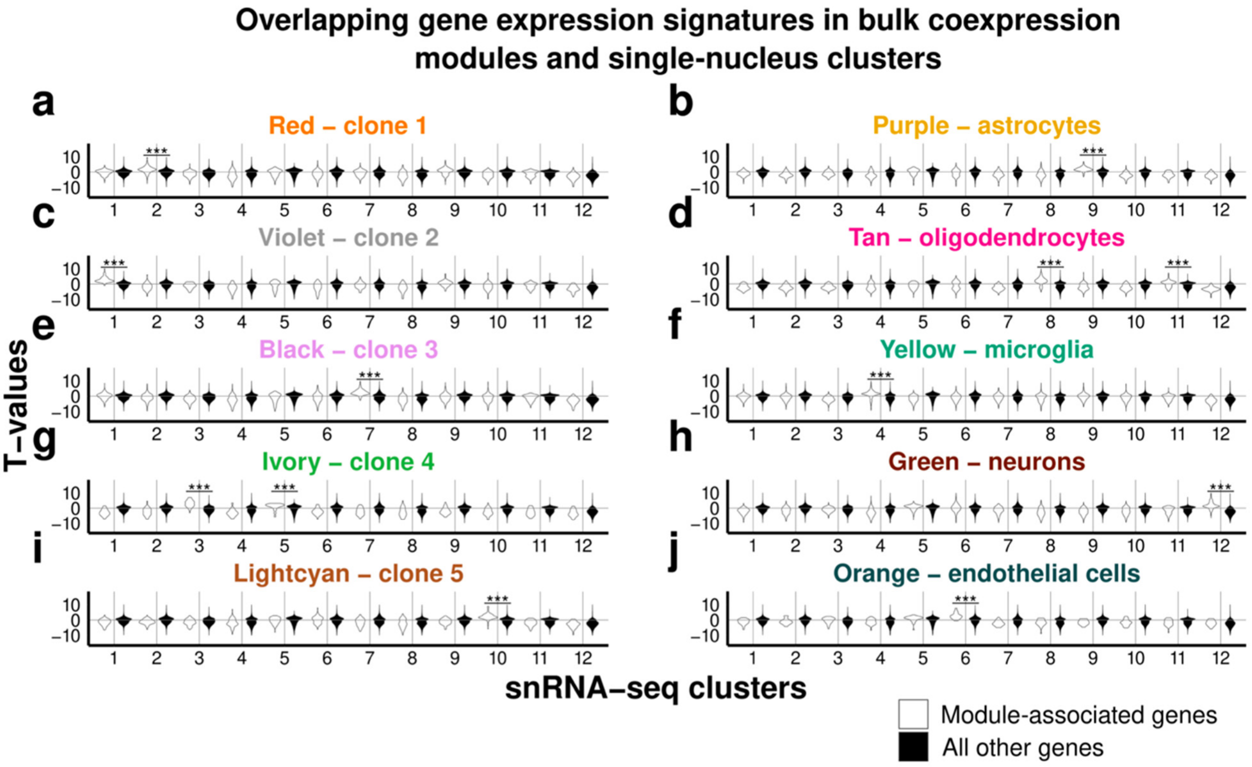

Figure S7. | Bulk coexpression module genes map definitively onto single-nucleus clusters. a-j) Modules of coexpressed genes from bulk tumor sections (n = 90) that were most strongly associated with specific clones (Figure 5d-g) or nonmalignant cell classes (Figure S5a-d) were evaluated for differential expression in each snRNA-seq cluster vs. all other clusters (white distributions: t-test results for all module genes). Genes that were not associated with each module were evaluated in the same fashion (black distributions), and a one-sided Wilcoxon rank-sum test was used to determine whether module genes were significantly upregulated in a given snRNA-seq cluster relative to all other genes (*** = P < 1e-10).

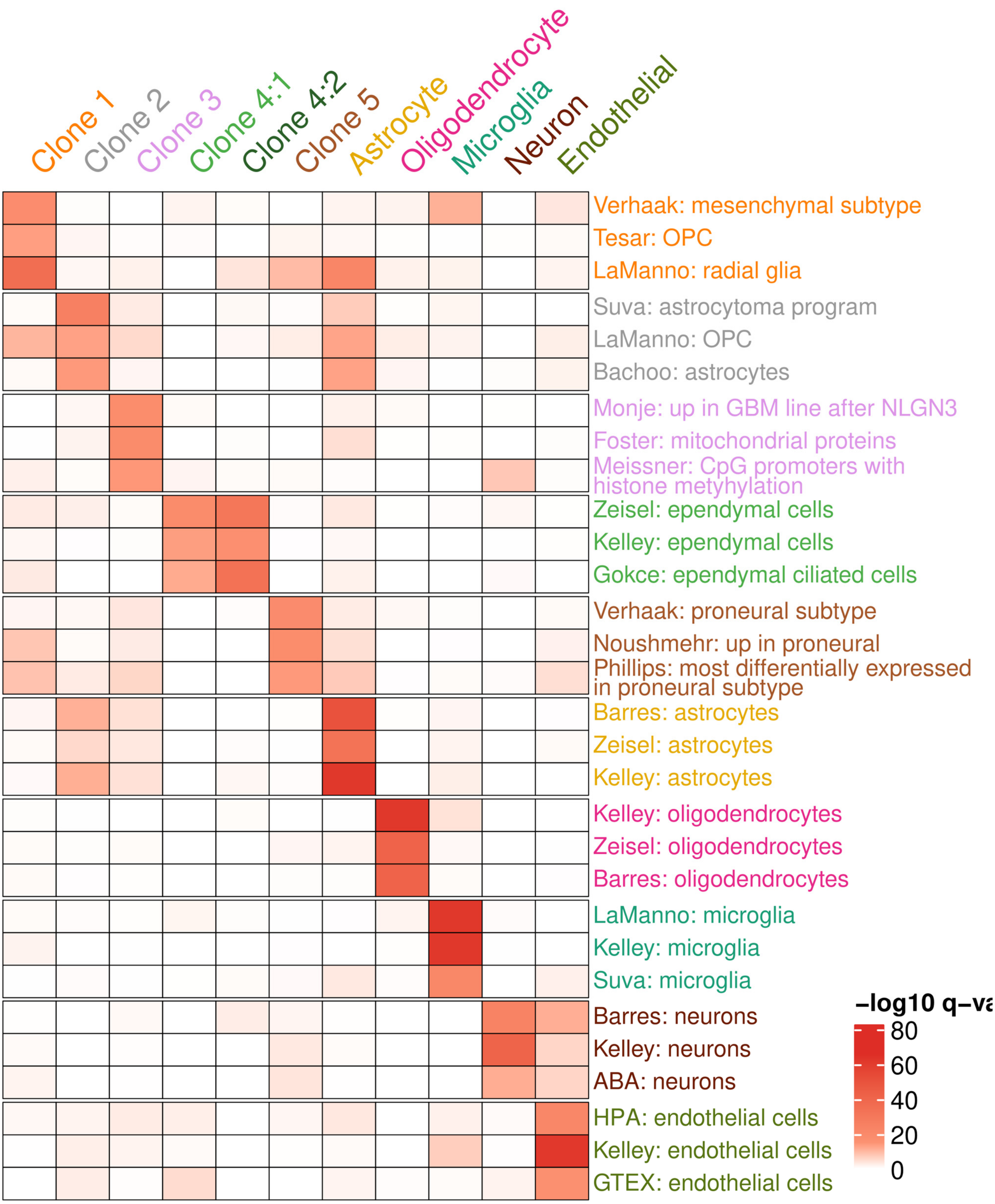

Figure S8. | Gene set enrichment analysis supports the functional distinctness of snRNA-seq clusters. Clustered heatmap of FDR-corrected p-values (q-values) from one-sided Fisher's exact tests comparing featured gene sets with genes that were significantly upregulated (FDR < .05) in each snRNA-seq cluster vs. all other clusters by the one-sided Wilcoxon rank-sum test.

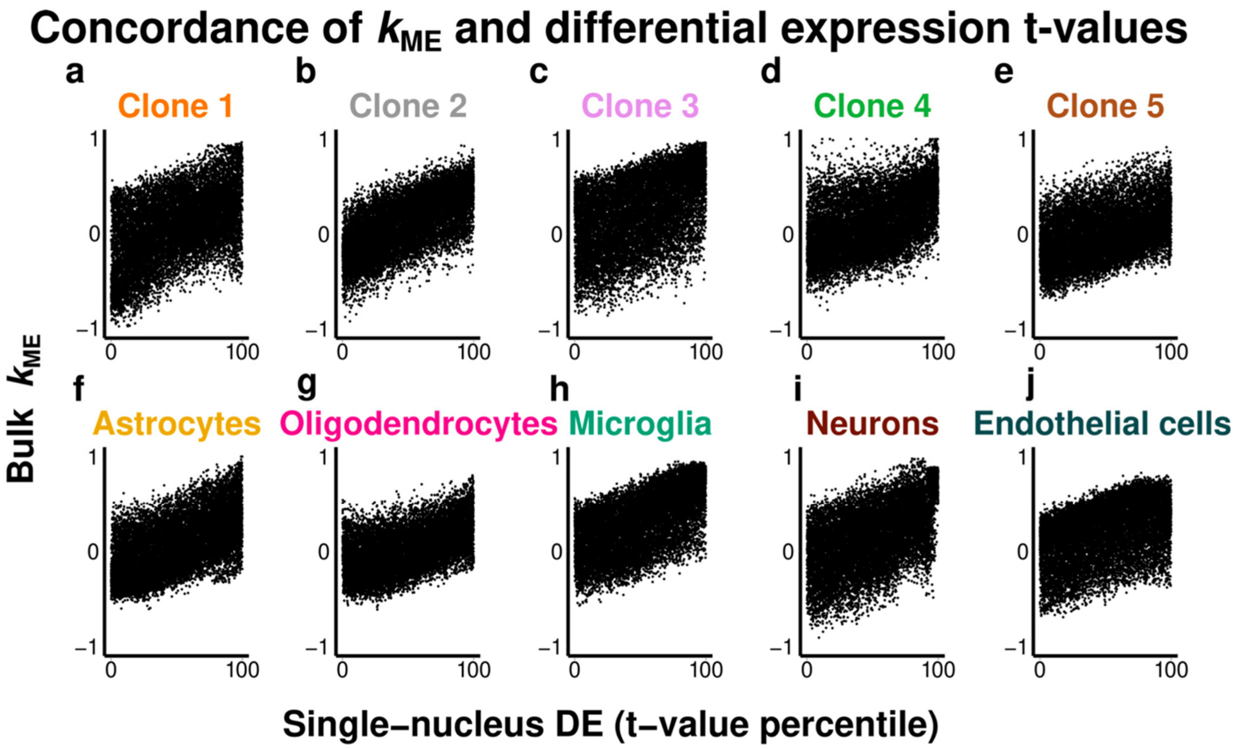

Figure S9. | Concordance of k_{ME} and differential expression t-values from bulk and single-nucleus experiments. a-j) Differential expression (DE) t-values (calculated by t-test for all genes between each snRNA-seq cluster and all other clusters) largely predict the extent to which gene expression patterns are correlated (k_{ME} values) to the bulk coexpression modules most strongly associated with each clone or nonmalignant cell type.
